# Trans-umbilical single-incision laparoscopic trans-abdominal pre-peritoneal hernioplasty of inguinal hernia by self-made glove port

**DOI:** 10.1097/MD.0000000000021787

**Published:** 2020-08-21

**Authors:** Qi-long Chen, Ke Chen, Di-yu Huang, Yu Pan, Jia-fei Yan, Xian-fa Wang, Xiao-yan Cai

**Affiliations:** Department of General and Minimally Invasive Surgery, Sir Run Run Shaw Hospital, College of Medicine, Zhejiang University, Hangzhou, Zhejiang, China.

**Keywords:** herniorrhaphy, inguinal hernia, laparoscopy, self-made, single-incision, TAPP

## Abstract

Laparoscopic inguinal herniorrhaphy has been well established for the management of primary and recurrent inguinal hernias. Single-incision laparoscopic surgery (SILS) has now been accepted as a less invasive alternative to conventional laparoscopic surgery. However, commercially available access devices for SILS had disadvantages such as rigidness and crowding. This series aimed to analyze the feasibility and safety of single-incision laparoscopic trans-abdominal pre-peritoneal hernioplasty (SILS-TAPP) by applying our self-made device for managing inguinal hernia.

We collected and reviewed the medical records of patients who received SILS-TAPP using a self-made glove-port device between January 2014 and January 2016. All operations were performed by the same surgical team. The demographics and intra- and perioperative outcomes were evaluated.

SILS-TAPP was successfully performed in 105 patients (131 inguinal hernia repairs). No major intra- and postoperative morbidities were encountered, and no conversion to a conventional 3-port approach or open surgery was required. The mean operative time was 73.5 min and the mean postoperative hospital stay was 2.1 days. Three minor short-term complications were noted, which were resolved without surgical intervention. One recurrence was diagnosed during follow-up and treated using a second TAPP procedure.

SILS-TAPP was shown to be a feasible, safe procedure in patients with an inguinal hernia. A simple self-made glove-port device was proven as a practical method of SILS-TAPP.

## 1 Introduction

Minimally invasive surgery (MIS) has been the main direction established in terms of surgical development in the 21st century.^[1]^ Laparoscopic inguinal herniorrhaphy (LIH) was first reported in the early 1990s and has grown in popularity compared with open inguinal herniorrhaphy because of its advantages such as pain reduction and rapid patient recovery.^[2–4]^ Laparoscopic trans-abdominal pre-peritoneal hernioplasty (TAPP) and laparoscopic total extraperitoneal hernioplasty (TEP) are the two most common procedures.^[5]^ However, the learning curve for TEP is longer and steeper than that for TAPP because of the pre-peritoneal view with which the surgeon is not accustomed and the limited working space.^[6]^ Traditionally, the operation involves inserting three ports: camera port below the umbilicus and two ports bilaterally that is lateral to the rectus muscle, resulting in three surgical scars.^[7]^ Efforts have been made to reduce the number of trocar- and port-site scars and improve cosmesis. Nowadays, single-incision laparoscopic surgery (SILS) has become increasingly popular because of its potential benefits in terms of recovery speed, pain reduction, and better cosmesis compared with conventional laparoscopic surgery.^[8,9]^ Laparoscopic trans-umbilical single-incision TAPP (SILS-TAPP) was first reported by Rahman and John in 2010.^[10]^ Specifically developed access devices are needed for the introduction of trocars and instruments in SILS.^[11]^ However, most commercially available access devices are rigid and have only one access point, which may hamper dissection due to its instrument crowding.^[11]^ We have developed a simple glove-port device using a commercially available wound protector and a surgical glove.^[12]^ The current study aimed to determine whether our self-made access device could be generally applied in SILS-TAPP, evaluate the surgical outcomes, and summarize our experiences.

## 2 Materials and methods

### 2.1 Patients

The study was approved by the Ethics Committee of Zhejiang University. A total of 105 patients underwent an elective laparoscopic SILS-TAPP at the Sir Run Run Shaw Hospital from January 2014 to January 2016. All patients who were diagnosed preoperatively with inguinal hernia were included. The exclusion criteria were as follows: (1) age of <20 years, (2) acute bowel incarcerated hernia, (3) previous retroperitoneal surgery such as for a recurrent hernia after laparoscopic hernioplasty or prostatectomy, or (4) compromised cardiopulmonary function. All SILS-TAPP procedures were performed by the same surgical team, which has an extensive experience of TAPP, TEP, and other MIS over the last 5 years.^[12,13]^ All patients provided informed consent for the surgical procedure. The evaluated variables were the patients’ demographics, surgical indications, intraoperative details (conversion, operative time, and estimated blood loss), and short-term outcomes (pain score, complication, and postoperative hospital stay). The pain score was graded using the visual analogue scale (VAS). The VAS was checked at postoperative 24 hours. Postoperative morbidity was graded using the Clavien**-**Dindo classification.^[14]^

### 2.2 Self-made glove-port device

**Figure 1:**
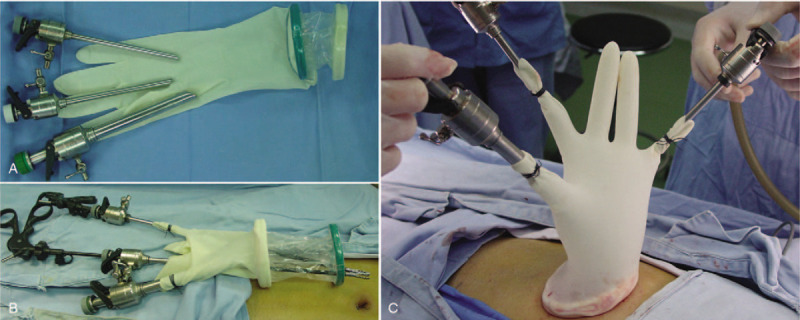
Illustrations of the Self-made glove-port device. (A and B) A surgical glove was attached to a wound protector, and trocars were introduced through the fingers of the gloves. (C) The device allowed greater movement feasibility of the instrument.

In brief, the device was constructed using a commercial wound protector (VIPA60, Changzhou, China), surgical glove, and three trocars (5, 5, 10 mm). Trocars were introduced through the little finger, thumb, and middle finger of the glove, respectively, and then strengthen by silk ligature to make the upper part of the device. The bottom ring of the wound protector, which was wrapped by the glove without suture, was placed into the abdominal cavity and supported the umbilical incision, while the top ring could turn over together after overlapping with gloves (Fig. 1).

### 2.3 Surgical procedure

**Figure 2:**
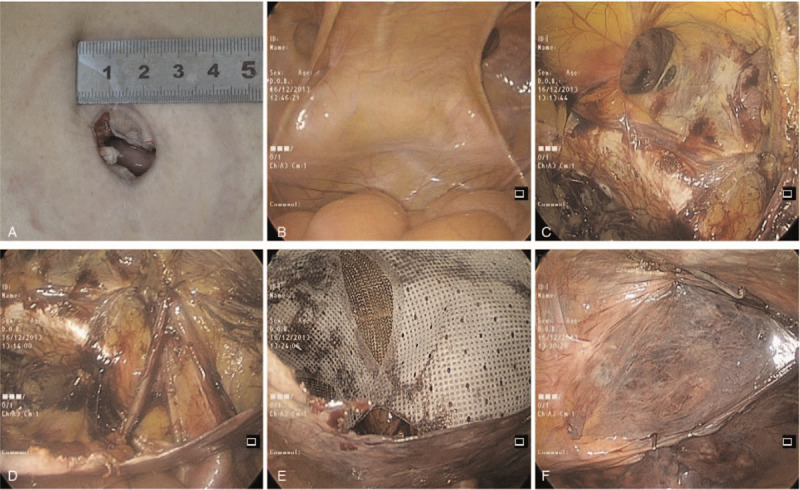
Intraoperative laparoscopic photographs. (A) Umbilicus incision was made. (B) Exposure of the hernia defect. (C) Exposure of the pubic symphysis and Cooper's ligament. (D) Visualization of the spermatic cord and myopectineal orifice. (E) The mesh was placed to overlap the hernia opening. (F) Closure of the peritoneal defect with suture.

The patient was placed in a supine position under general anesthesia. The table was given a 30° Trendelenburg tilt, and the side on which the surgery was to be performed was tilted up by 30° to allow the small bowel loops to fall back from the groin region. A 2-cm-sized trans-umbilical vertical incision was made (Fig. 2A), and the peritoneal cavity was entered (Fig. 2B). The wound retractor was set up through the trans-umbilical incision, and the surgical glove was fixed to the wound retractor and served as a single port. The trans-umbilical skin incision was stretched and elongated by the self-made glove-port device; therefore, it was possible to insert three instruments through the trans-umbilical skin incision. The laparoscope with a 30° angle was inserted through the 10-mm trocar, and laparoscopic instruments were inserted through the two 5-mm trocars. A CO_2_ pneumoperitoneum was produced with a maximal pressure of 12 mmHg so that the glove apparatus could be quite stable, and we could avoid the balloon effect of the glove device. The surgeon stood on the opposite side of the hernia, while the first assistant handling the camera stood beside the patient's opposite shoulder. The basic operative steps of single-incision TAPP mesh hernioplasty are similar to those of conventional laparoscopic TAPP mesh hernioplasty. The peritoneum was incised over the hernia and extended laterally using electrocautery. The rectus abdominis muscle, horizontal pubic ramus, Hesselbach's triangle, Cooper's ligament, and iliopubic tract were exposed. A gap was carefully created between the hernial sac and spermatic cord (round ligament in women) at approximately 1 to 2 cm away from the neck of the hernial sac. The hernial sac was reduced meticulously and carefully, preserving the inferior epigastric vessels and vas deferens using conventional endoscopic graspers and endodissector. The pubic symphysis was clearly defined medially (Fig. 2C). The sac was completely dissected, and the myopectineal orifice was freed from tissue. The fascia spermatica and nerves located in the parietal compartment were spared (Fig. 2D). The vas deferens and gonadal vessels were parietalized sufficiently to allow the placement of a 15 × 10-cm mesh. For the insertion of the mesh into the peritoneal cavity, the glove was removed from the wound retractor, and the mesh was introduced. The glove was refixed to the wound retractor. The mesh was placed medially across the pubic symphysis and laterally up to the lateral end of iliopubic tract (Fig. 2E). The mesh was fixed to Cooper's ligament medially and at the superolateral angle using an ENDOPATH Multifeed Stapler (EMS, Ethicon Endo-Surgery Inc., Cincinnati, OH, USA). The peritoneum was then fixed over the mesh using EMS or a continuous 3-0 Vicryl suture (Fig. 2F). Pneumoperitoneum was released under vision. The fascial and skin incisions were meticulously closed layer by layer.

## 3 Results

**Figure 3:**
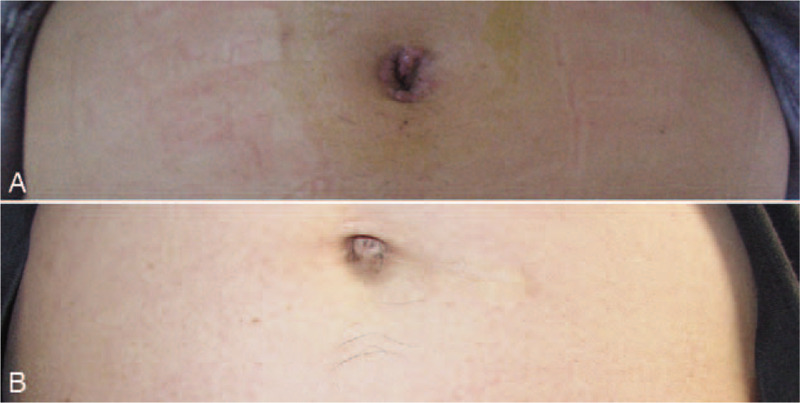
Postoperative view of umbilicus wound. (A) 7 days. (B) 3 months.

Patients’ demographics and hernia characteristics are summarized in Table 1. A total of 131 inguinal hernia repairs, in which 14 (10.7%) were recurrent hernias in 105 patients were included. Twenty-six of the patients had bilateral hernia and the remaining had unilateral hernia. The mean age of the patients was 51.4 years (range, 19–73 years), and the male-to-female ratio was 12.1:1 (97 male). Their mean body mass index (BMI) was 23.1 kg/m^2^ (range, 17.6–29.2 kg/m^2^). Intraoperative outcomes and postoperative recoveries are presented in Table 2. No conversion from SILS-TAPP to a conventional 3-port approach or open surgery was necessary. There was no major bleeding resulting from injury to the external iliac or main inferior epigastric vessels, and blood transfusion was not needed. No other intraoperative complications such as injury of the urinary bladder and bowel were observed. The mean postoperative hospital stay was 2.1 days (range, 1–4 days). The median value of the VAS score was 3 (range, 1–5) 24 hours after surgery. Perioperative mortality, calculated within 90 days after surgery, was not observed. Morbidity occurred in 4 of the 105 patients with an overall morbidity rate of 3.8%: one patient had wound infection of the umbilical port that was treated successfully by wound dressing in the outpatient department; one urinary tract infection was diagnosed and treated using by antibiotic therapy; one patient needed a drainage of a large local hematoseroma 48 hours postoperatively. All short-term postoperative complications were resolved without surgical intervention. Postoperative cosmesis effect was excellent (Fig. 3). The median follow-up period was 58 (48–72) months. One hernia recurrence was diagnosed 13 months after surgery. The patient was treated using a second TAPP procedure and without additional hernia relapse during the follow-up period. None of other patients reported neuralgia, incisional hernia or any other significant problem during their follow-up.

**Table 1:**
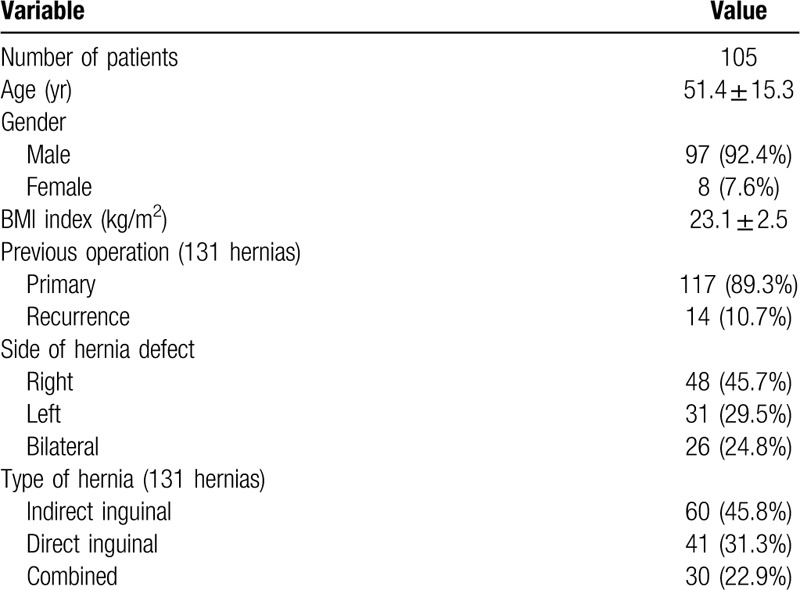
Patients’ demographics and hernia characteristics.

**Table 2:**
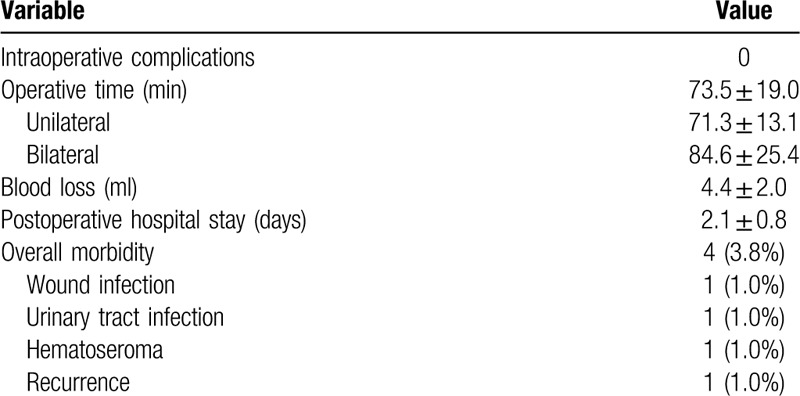
Intraoperative outcomes and postoperative recoveries.

## 4 Discussion

MIS has been widely considered superior to open surgery in various fields. LIH has made significant strides and has become the first choice for inguinal hernia repair in many canters after a decade of experience.^[15]^ For better cosmesis, several surgeons have attempted to reduce the port numbers and sizes during laparoscopic surgery to reduce the appearance of a scar.^[16]^ Till date, several studies and meta-analysis showed SILS to be superior over multiport laparoscopic surgery mainly in terms of postoperative pain and cosmetic result.^[8,9,17,18]^ Tanoue et al reported SILS-TAPP for 202 groin hernias, in which the operation time was 92 min for the unilateral hernias and 135.7 min for the bilateral hernias, and found that the overall morbidity was 8.2%, the average postoperative stay was 6.7 days, and the postoperative pain was short-lived.^[19]^ Commercially available access systems offer immovable trocar sites. Instrument crowding may thereafter commonly occur when access devices with immovable trocar sites are used.^[20]^ Curved instruments could be required, but this may eventually increase the total cost.^[20]^ Our self-made glove-port device was initially invented to overcome this inflated cost. Our series showed a 100% success rate of SILS-TAPP, performed with our self-made glove-port device and conventional instruments, and none of the patients required any additional port placement. No conversion to a conventional 3-port approach or open surgery was required. There was no major bleeding resulting from injury to the external iliac or main inferior epigastric vessels. Postoperative pain was minimal, which was consistent with other studies.^[19,21,22]^ Cosmetic effect is the major benefit, but the underlying risk of port-site hernia should be additionally investigated. Furthermore, long-term follow-ups are warranted.

There are two commonly used LIH methods: TAPP and TEP.^[21,22]^ Using TAPP, the great intra-peritoneal working space makes it easier and safer to perform laparoscopic operations. TAPP is advantageous as it allows to simultaneously evaluate and manage the concurrent hernia in contralateral side and easily assess the type and contents of hernia under the guidance of a laparoscope. When TEP is performed, the dissection balloons are quite expensive in addition to the restricted working space. TAPP is preferred in repairing laparoscopic groin hernia in our center. With regard to SILS, it shows cosmetic benefit as it is conducted in a trans-umbilicus approach; in this way, the operative wound is “invisible”. However, SILS has its own disadvantages: (1) A confined and limited working space, (2) In-line position of the laparoscope, (3) close proximity of the working instruments with limited triangulation, and (4) restricted range of motion of the instruments.^[19]^

We have summed up the following experience in practice. (1) Most commercially available access devices are designed for laparoscopic cholecystectomies and appendectomies, which were unfit for herniorrhaphy and costly.^[23]^ Goo et al reported SILS-TAPP using SILS Port System (Covidien, Norwalk, CT) and found that the system had a small operating hole, which made it difficult to place the mesh; therefore, it was necessary to replace the SILS port into a 12-mm trocar to place the mesh into the abdominal cavity.^[24]^ Roy and De also reported SILS-TAPP utilized the conventional laparoscopic instruments that three trocars were placed into the fascia layer through an approximately 2-cm umbilical incision the single skin incision 2 cm around the umbilicus. Unfortunately, the umbilical scar was obvious, and the cosmesis was undesirable.^[25]^ Our easy-sampling system will not increase the financial burden of the patient and is particularly suitable to junior hospitals where hernia is a common disease (Fig. 3). (2) One of the most challenging factors for SILS in attaining widespread use is the additional learning curve required for this technique.^[26]^ The commercially rotatable single-port device that was designed curved need a cross-operation,^[27]^ which could be avoided by our easy-mastered system, thus reducing the operative difficulty and shorting the learning curve. (3) Our system allowed greater movement feasibility of the instrument. Gloves have favorable expansion and scaling properties; as a result, the operator can control the horizontal or rotational free transposition of the device for separation operation to obtain the satisfactory “operational triangle.” The core channel for the instrument import and export in the commercial devices has been simplified from the originally independent and adjacent ports to a single open channel, which facilitates device free transposition and rotation, and contributes to the flexible adjustment of laparoscope and instrument position. In addition, it is easy to maintain stability when operating as the devices are light; such design gives the operation more flexibility. (4) For patients with narrow and deep-set umbilicus, the design adopts a zigzag skin incision, which can improve the short diameter and narrow straight-line incision and obtain the relatively satisfying operating space.^[28]^ For patients with a relatively flat and valgus umbilicus, the short straight-line incision can obtain satisfactory space, and better cosmetic effect is achieved after incision suturing. (5) The incision retractor has obvious incision expanding effect, which can expand the original length of incision from 2.0 to 2.5 cm, and maximize the incision potential to allow for the free pass of the device. Our self-made device is suitable for all body types (fat or thin) because the wound protector can be rolled for all kinds of body sizes of patients. More important is that any type of trocar can be used for our self-made glove-port system. (6) Sharp dissection should be appropriately adopted to cut open the peritoneum and dissociate the pre-peritoneal space. During SILS-TAPP, there is certain obstruction between the devices, and it is difficult to substantially separate and advance the device. Consequently, cutting open the peritoneum intraoperatively and dissociating the pre-peritoneal space should be conducted using scissors or electrocoagulation hook. Attention should be paid to dissociate point by point under the direct vision with a laparoscope and then from the point to a line and from the line to the complete myopectineal orifice plane. However, we realize that there are still drawbacks in closing the peritoneal incision, which is the extremely high difficulty in suturing under the SILS approach. We initially attempted to adopt an intracorporeally hand-sewn stitch for closing the peritoneal incision, but the procedure was time- and effort-consuming. In addition, it was difficult in tying a knot, and clips were needed to clamp the stitches. Staples (EMS) were thus used in the following cases to simplify the closure. In contrast, essentially larger trans-umbilical incision in SILS is more likely to increase the incidence of incisional hernias. Meticulously closing the incision layer by layer is extremely crucial. Although no umbilical port-site hernia was observed in this series, the potential risks still require further studies and deliberations on a long-term follow-up.

Admittedly, this study is limited by its small sample size, enrollment of patients with different statuses, absence of a control group, and lack of long-term satisfaction and cosmetic outcomes. Moreover, whether the advantages in cosmesis and morbidity could overcome the technical difficulties of SILS need to be verified. Therefore, randomized, comparative studies with large sample sizes are necessary to draw definitive conclusions regarding the role of our self-made glove-port device in SILS-TAPP for inguinal hernia repair.

## 5 Conclusion

In conclusion, we successfully performed SILS-TAPP using a self-made glove-port device with a flexible fulcrum to allow a single-port access surgery to be performed. Preliminary results appear safe, feasible, and reproducible in terms of perioperative outcomes. Short-term cosmetic improvement is noticeable. Nevertheless, further analyses are mandatory to validate our findings.

## Author contributions

**Conceptualization:** Ke Chen.

**Data curation:** Di-yu Huang, Yu Pan.

**Funding acquisition:** Xiao-yan Cai.

**Investigation:** Ke Chen, Yu Pan.

**Methodology:** Xiao-yan Cai.

**Supervision:** Xian-fa Wang, Xiao-yan Cai.

**Writing – original draft:** Jia-fei Yan.

**Writing – review & editing:** Qi-long Chen, Xiao-yan Cai.
